# Construction and validation of molecular subtypes of coronary artery disease based on ferroptosis-related genes

**DOI:** 10.1186/s12872-022-02719-1

**Published:** 2022-06-22

**Authors:** Lina Ding, Fei Long, Dan An, Jing Liu, Guannan Zhang

**Affiliations:** Department of Cardiology, The Third Affiliated Hospital of Jinzhou Medical University, No. 2, Section 5, Heping Road, Linghe District, Jinzhou, 121000 Liaoning China

**Keywords:** Coronary artery disease, Ferroptosis, Molecular subtypes, Non-negative matrix factorization (NMF)

## Abstract

**Background:**

This study aims to construct a reliable diagnostic model for coronary artery disease (CAD) patients and explore its potential mechanism by consensus molecular subtypes of ferroptosis-related genes.

**Methods:**

GSE12288 and GSE20680 were downloaded from Gene Expression Omnibus database. CAD patients were divided into different molecular subtypes according to the expression level of ferroptosis-related genes. Then, the distribution of differentially expressed genes, functional annotations and immune infiltration cells between the two subtypes were compared. Finally, a prognostic model of ferroptosis-related genes in CAD was constructed and verified.

**Results:**

Two different molecular subtypes of CAD were obtained according to the expression level of ferroptosis-related genes. Then, a total of 1944 differentially expressed genes (DEGs) were found, among which, 236 genes were up-regulated and 1708 genes were down-regulated. In addition, 43 DEGs were ferroptosis-related genes. Functional enrichment analysis showed that these DEGs between two subtypes of CAD were mainly enriched in immune-related pathways and processes, such as T cell receptor, mTOR, NOD-like receptor and Toll-like receptor signaling pathways. We also found that 21 immune cells were significantly changed between two subtypes of CAD. The LASSO method was performed to identify and construct the 16 ferroptosis-related genes-based diagnostic signature. Diagnostic efficiency of diagnostic signature measured by AUC in the training set and validation cohort was 0.971 and 0.899, respectively.

**Conclusions:**

This study contributes to a more comprehensive understanding of the mechanism of ferroptosis-related genes in CAD.

**Supplementary Information:**

The online version contains supplementary material available at 10.1186/s12872-022-02719-1.

## Background

Coronary artery disease (CAD), a multifactorial and complex disease, is considered to be one of the most dangerous cardiovascular diseases. Based on the epidemiological studies, the number of CAD patients will rapidly increase over the next decade [1]. At present, diagnosis means of CAD are mainly coronary artery contrast CT and cardio-angiography [2]. Nonetheless, these diagnostic techniques require specialized medical centers and experienced cardiologists, limiting their routine use in clinical practice. Thence, the development of new biomarkers for early diagnosis of CAD is urgently needed. In recent years, mRNA expression in peripheral blood has been reported to be associated with multiple diseases, such cancer [3], hypertension [4] and diabetes [5]. However, the diagnostic value of mRNA in peripheral blood sample in CAD patients is still unclear.

Iron is a basic nutrient element in the human body and is essential for biological processes, including cell metabolism, growth and proliferation. Iron-induced oxidative stress is associated with a variety of pathological conditions, such as CAD, heart failure, cardiomyopathy and atherosclerosis [6, 7]. Ferroptosis is an iron-dependent programmed cell death process that different from other forms of cell death. Recently, the mechanism of ferroptosis has been the focus of researchers. Studies have shown that ferroptosis plays an important role in the pathogenesis of various tumors, including lung cancer, breast cancer, colorectal cancer, bladder cancer and hepatocellular carcinoma [8]. It has also been suggested that ferroptosis is associated with the mechanism of cell death in cerebral hemorrhage and ischemia–reperfusion injury [9, 10]. Recently, a study has reported that ferroptosis is a significant form of cell death in cardiomyocytes [11]. However, the role of ferroptosis in CAD remains largely unknown.

With the development of microarray technology and high-throughput sequencing technology, it provides an opportunity to further understand the genetic and molecular basis of CAD [12, 13]. However, most of the current studies focus on the differences between CAD and normal controls, and less attention is paid to the differences between different subtypes of CAD. In cancer research, tumor samples are usually divided into several molecular subtypes according to gene expression patterns, which can reveal the heterogeneity between tumors and predict clinical endpoints [14, 15]. CAD has clinical heterogeneity, and the identification of CAD molecular subtypes based on gene expression pattern may also provide a new way for the diagnosis and treatment of CAD.

In this study, ferroptosis-related genes in CAD were obtained and analyzed. Then, CAD samples were clustered into two molecular subtypes based on the expression of ferroptosis-related genes. In addition, differentially expressed genes (DEGs), functional enrichment analysis and characteristics of immune cell infiltration between two subtypes of CAD were analyzed. Furthermore, a ferroptosis-related diagnostic model was established based on mRNA expression profiles of CAD patients from GSE12288 dataset and validation in GSE20680 dataset. This study may provide a theoretical basis for further studies in this field.

## Materials and methods

### Data acquisition

Two datasets (GSE12288 and GSE20680) were downloaded from Gene Expression Omnibus (GEO) database (https://www.ncbi.nlm.nih.gov/geo/). In total, 110 CAD and 112 normal controls peripheral blood samples were enrolled in GSE12288 dataset (platform: GPL96 [HG-U133A] Affymetrix Human Genome U133A Array). This dataset was selected according to Duke CAD index (CADi). CADi is a validated angiographical measure of the extent of coronary atherosclerosis that correlates with outcome. GSE20680 was based on the platform of GPL4133 Agilent-014850 Whole Human Genome Microarray 4 × 44 K G4112F and contained peripheral blood sample from 143 CAD and 52 normal controls. This dataset included 3 condition samples: Cases (2) are patients with ≥ 70% stenosis in > 1 major vessel or ≥ 50% stenosis in > 2 arteries; intermediates (1) are patients with luminal stenosis > 25% but less than 50%; controls (0) have luminal stenosis of ≤ 25%. All data processing was performed using R software (version 3.5.3). The GSE12288 dataset was used as the training set and the GSE20680 dataset was used as the verification dataset.

### Acquisition of ferroptosis-related genes

Ferroptosis-related genes were first downloaded from the FerrDb website (http://www.zhounan.org/ferrdb/index.html). The confidence levels of genes involved in ferroptosis were assigned to 4 degrees including validated, screened, predicted, and deduced. The species involved humans, mice, rats, and drosophila. Then the genecard database (https://www.genecards.org/) was searched with the keyword “ferroptosis” to supplement the ferroptosis-related genes list. A total of 156 ferroptosis-related genes were identified after removal of non-coding RNA (Additional file 4: Table S1).

### Consensus molecular subtyping with non-negative matrix factorization (NMF)

Afterwards, ferroptosis-related genes were performed in non-negative matrix factorization (NMF) clustering [16, 17]. R package NMF (version 0.21.0) was used to decompose gene expression matrix A. Matrix A was factorized into 2 nonnegative matrices W and H (i.e., A≈WH). Repeated factorization of matrix A was performed and its outputs were aggregated to obtain consensus clustering of CAD samples. The optimal number of subtypes was selected according to cophenetic, dispersion, and silhouette coefficients [18]. Wilcoxon’s rank sum test was used to examine the difference between CAD indexes and age among different subtypes.

### Identification of differentially expressed genes (DEGs) in different subtypes

In order to explore the biological differences between different subtypes, we used the "limma" R package to identify DEGs between different subtypes. False discover rate (FDR) value < 0.01, and |log2fold change| (|log2FC|) > 0.3 was considered statistically difference. At the same time, the "limma" R package was used to calculate the P value. P value represents the significance of gene expression differences between different subtypes. Subsequently, ggplot2 was used for volcanic maps. Heat maps were used to visualize DEGs.

### Functional enrichment analysis

Gene Ontology (GO) and Gene Set Enrichment Analysis (GSEA) were applied to disclose the biological function of DEGs. GO analysis of DEGs was performed using David 6.8 (https://david.ncifcrf.gov/tools.jsp). FDR < 0.05 was considered statistically significant. Then, GSEA was used to investigate the pathways enriched in the different subgroups utilizing the “clusterProfiler” R package. c2.cp.kegg.v7.0.symbols.gmt was selected as the gene set database. The cutoff criteria were set at FDR < 0.05.

### Single-sample gene set enrichment analysis (ssGSEA) in GSE12288 dataset

The ssGSEA algorithm was carried out to quantify the relative abundance of immune cell infiltration in each CAD sample. Enrichment score calculated by ssGSEA analysis was used to represent the relative abundance of immune infiltrating cells in each tissue sample. The wilcoxon test was used to calculate the significance of differences in immune cell infiltration between different subgroups. Heat map and box plot were used to compare the differences in the level of immune cells infiltration in different sample tissues.

### Development and validation of diagnostic models

Least absolute shrinkage and selection operator (LASSO) regression method was introduced to select the key genes for establishing diagnostic model for CAD. The “glmnet” package was used in LASSO regression analysis. This method shrinks coefficients toward zero, and eliminates unimportant terms entirely, thus reducing prediction error and minimizing overfitting. Then, diagnosis-associated DEGs with nonzero coefficients were elected to establish a diagnostic gene signature. The receiver operating characteristic (ROC) analysis was performed using R package pROC (version 1.15.3), and the area under the curve (AUC) was calculated to evaluate the accuracy of the model.

### Statistical analysis

R package (version 3.6.3; https://www.R-project.org) was used for all statistics. The wilcoxon test was used to calculate the significance of differences in immune cell infiltration, ferroptosis-related genes expression, CADi and age between clusterA and clusterA groups. Fisher accurate test was used to compare the proportion of Cases (2) patients in different subtypes. P < 0.05 was statistically significant.

## Results

### Characterization of two ferroptosis-related molecular subtypes

**Fig. 1:**
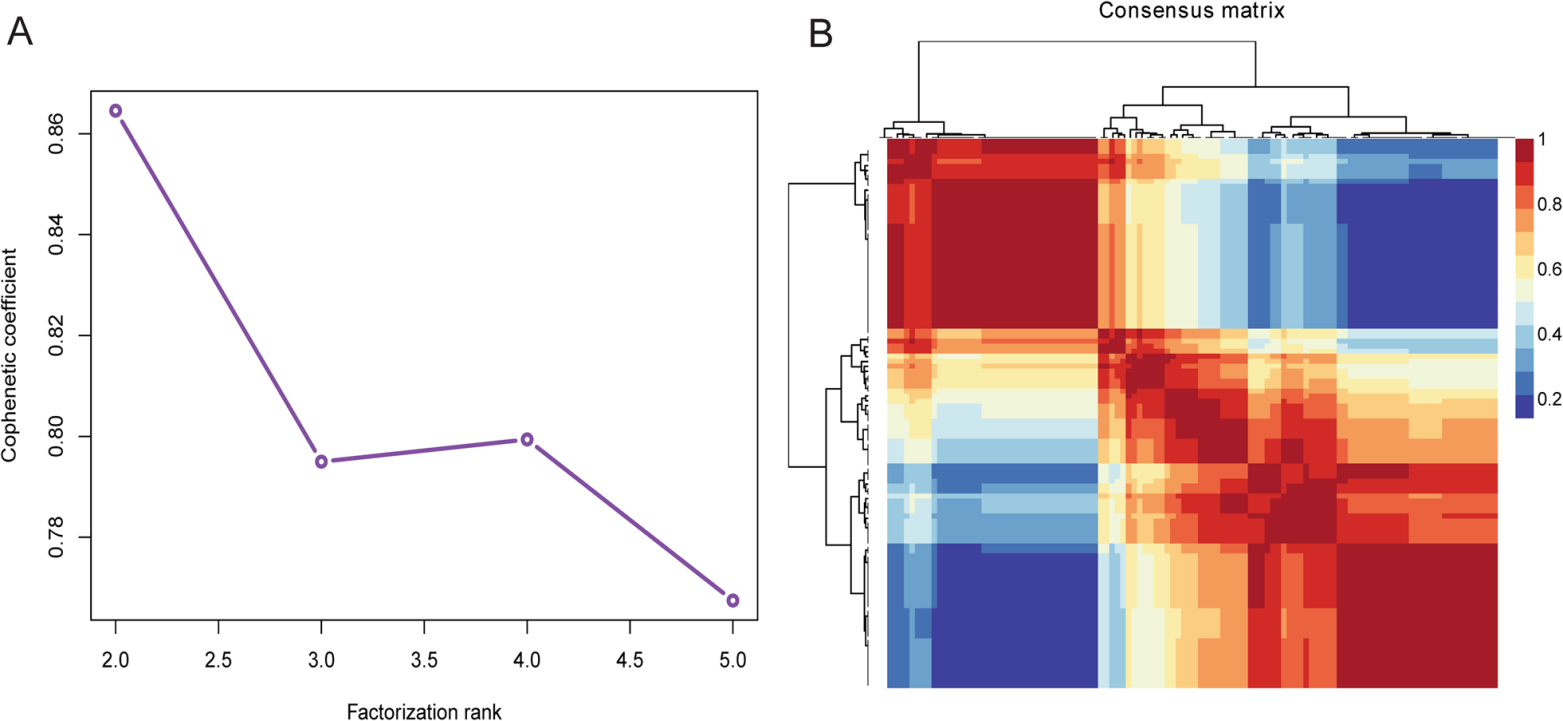
Identification of consensus clusters by ferroptosis-related genes. **A** The relationship between cophenetic and silhouette coefficients with respect to the number of clusters. **B** Consensus matrix heat map for k = 2

**Fig. 2:**
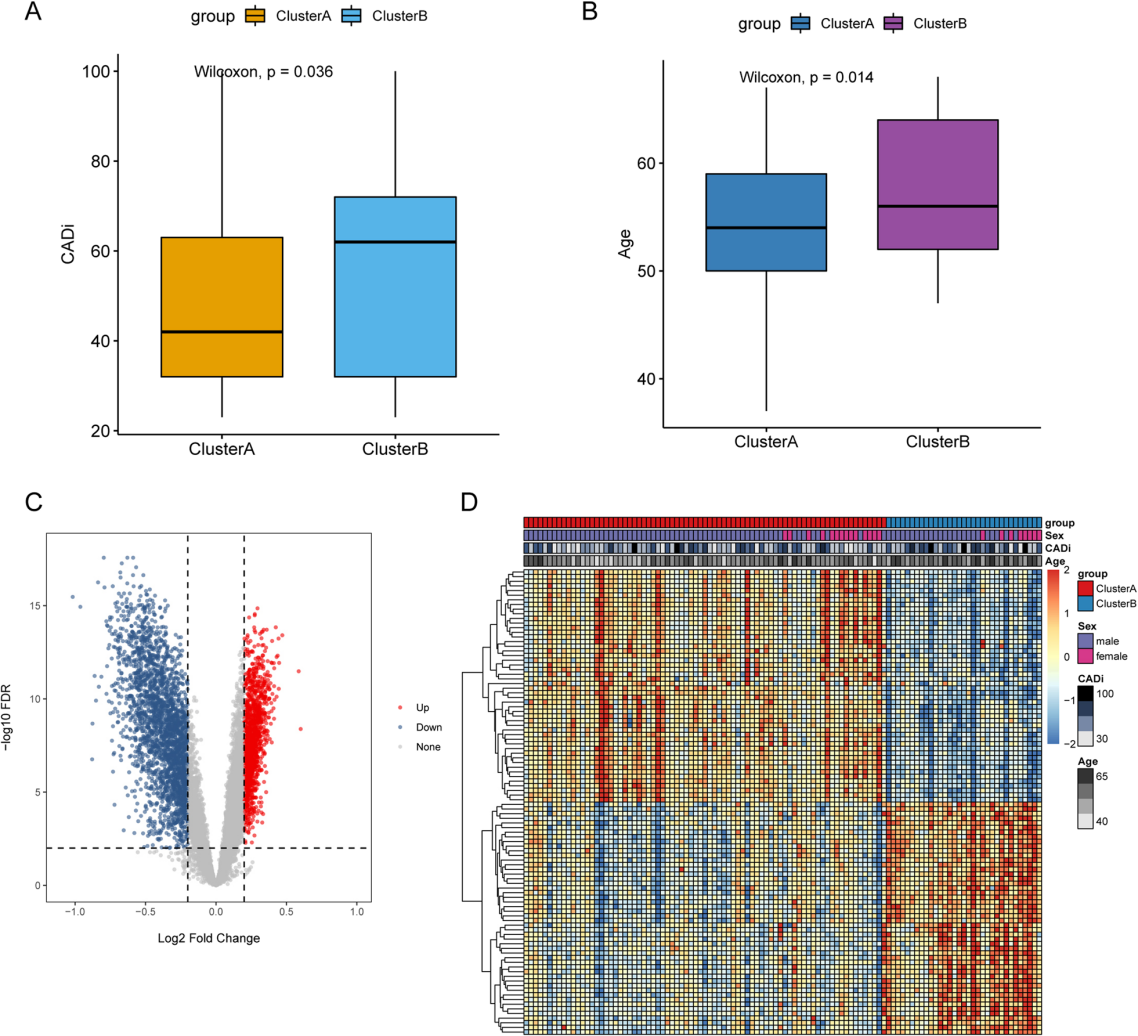
Characterization and DEGs of two ferroptosis-related molecular subtypes of CAD. **A** The difference of CADi between two subtypes of CAD. **B** The difference of age between two subtypes of CAD. (C) Volcanic maps between two subtypes of CAD. (D) The heat maps between two subtypes of CAD

According to the expression of 156 ferroptosis-related genes, 110 CAD samples were divided into subgroups by consensus clustering analysis of NMF package. The optimal k value was obtained based on the comprehensive correlation coefficient. When starting from k = 2, comprehensive correlation coefficient started to decrease (Additional file 1: Fig. S1 and Fig. 1A). The heat map showed a clear and sharp boundary, suggesting stable and robust clustering for the samples (Fig. 1B). Therefore, 110 CAD samples were clustered into two molecular subtypes clusterA (n = 77) and clusterB (n = 33). Furthermore, Wilcoxon test results showed that the CADi and age of patients in clusterB subgroup were significantly higher than those in clusterA subgroup (Fig. 2A, B). The above results showed that there were two different disease subtypes in CAD. Compared with clusterA, patients with clusterB subtype were older and more severely ill.

### Identification of DEGs and functional enrichment analysis between two subtypes of CAD

**Fig. 3:**
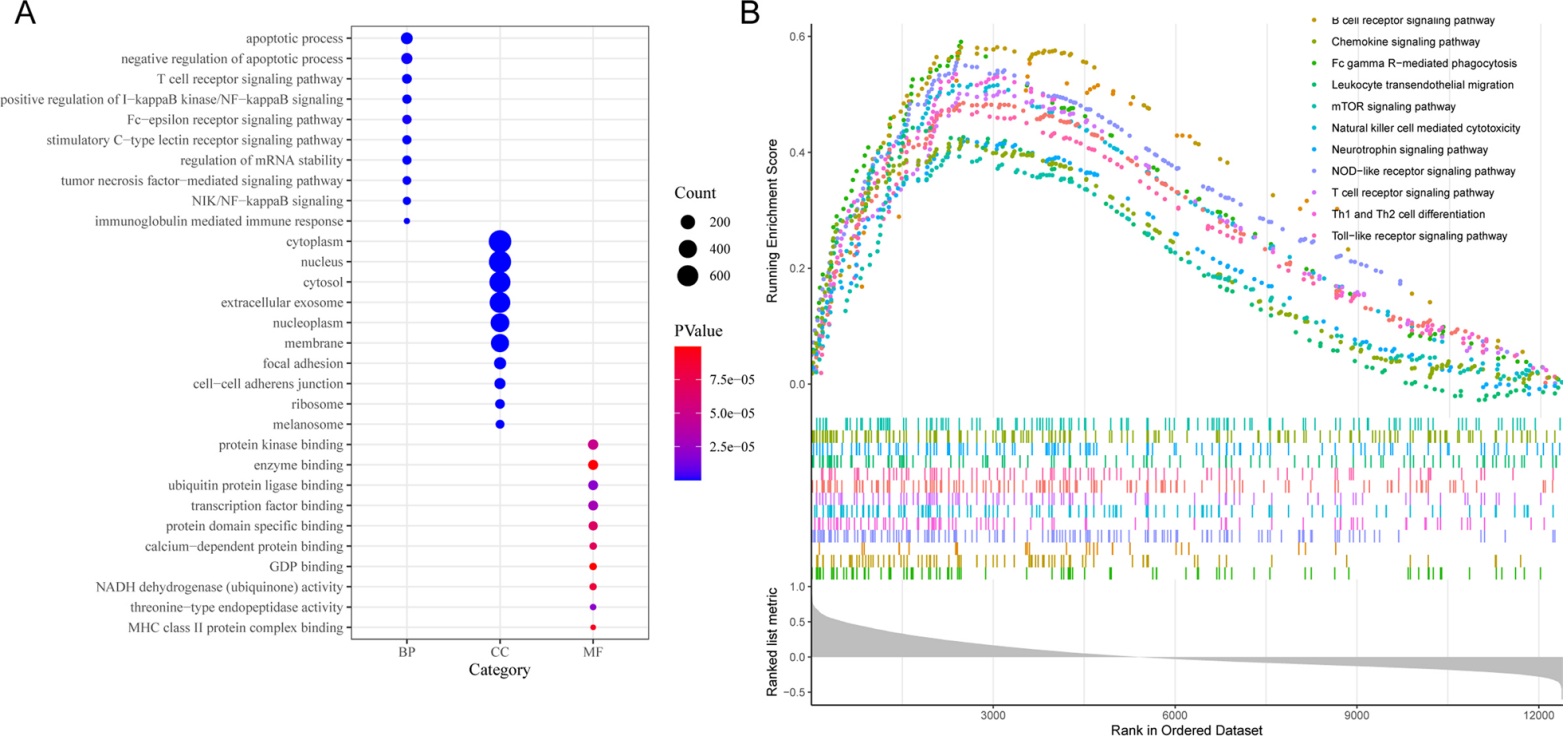
Functional enrichment analysis between two subtypes of CAD. **A** GO enrichment analysis. **B** GSEA analysis

In order to explore the differences between the two subtypes of CAD, we conducted a differential expression analysis in GSE12288 dataset. The "limma" R package was used to identify DEGs between different subtypes. A total of 1944 DEGs were found, among which, 236 genes were up-regulated and 1708 genes were down-regulated. Additionally, 43 DEGs were ferroptosis-related genes (Additional file 5: Table S2). The volcanic maps and heat maps is displayed in the Fig. 2C, D, respectively. GO enrichment analysis showed that these DEGs were mainly enriched in immune and inflammatory processes, including T cell receptor signaling pathway, NIK/NF-kappaB signaling, positive regulation of I-kappaB kinase/NF-kappaB signaling and immunoglobulin mediated immune response (Fig. 3A and Additional file 6: Table S3). The GSEA analysis revealed that DEGs were mainly enriched in B cell receptor signaling pathway, Chemokine signaling pathway, Leukocyte transendothelial migration, mTOR signaling pathway, Natural killer cell mediated cytotoxicity, Neurotrophin signaling pathway, NOD-like receptor signaling pathway, T cell receptor signaling pathway, Th1 and Th2 cell differentiation, Toll-like receptor signaling pathway (Fig. 3B and Additional file 7: Table S4).

### Characteristics of immune cell infiltration in two subtypes of CAD

**Fig. 4:**
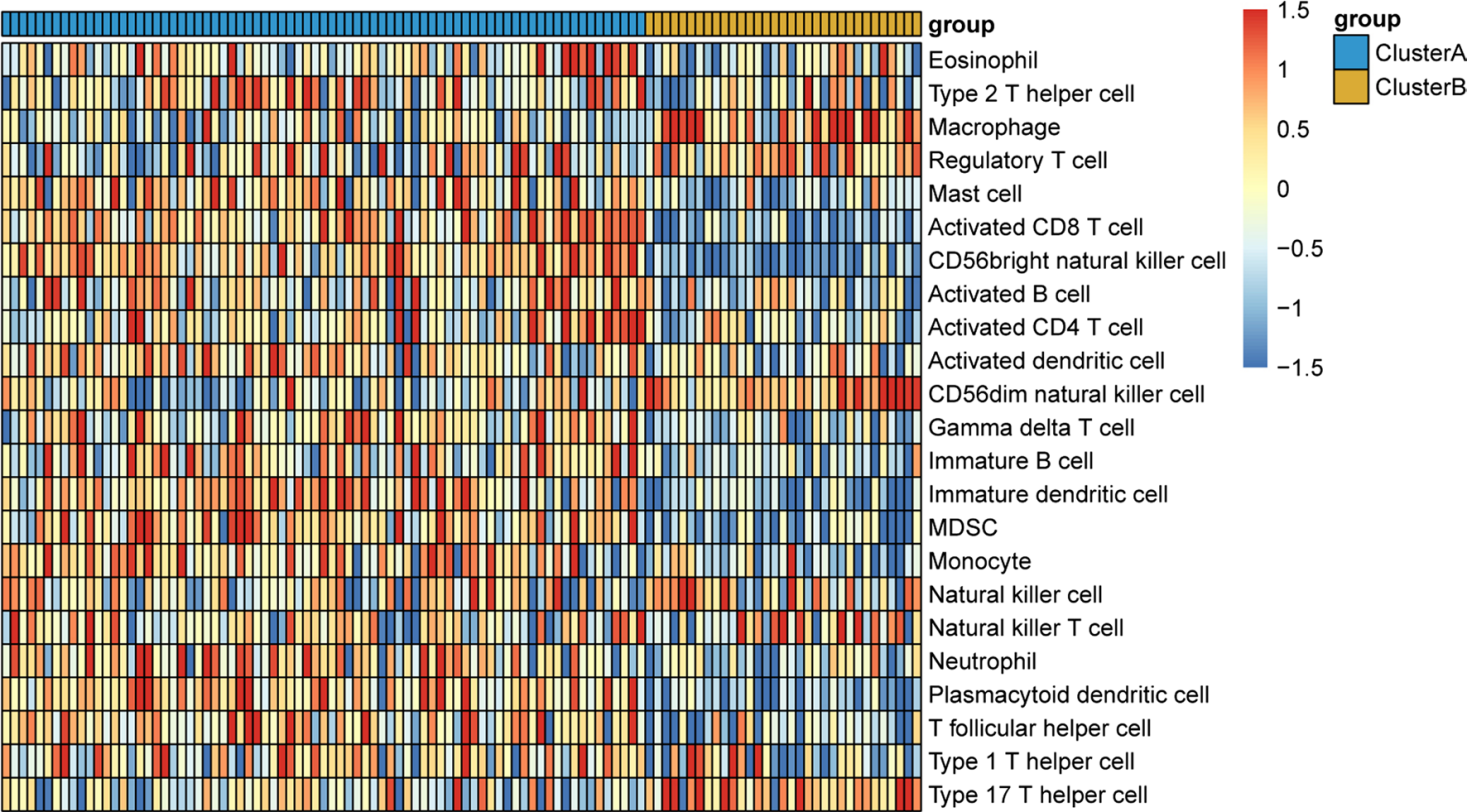
Heat map of immune infiltration cells between two subtypes of CAD

Heat map of immune infiltration cells between two subtypes of CAD is shown in Fig. 4. SsGSEA results indicated that except for Natural killer cell and Natural killer T cell, 21 other immune infiltration cells were significantly changed between two subtypes of CAD (Additional file 8: Table S5). Of which, Eosinophil, Type 2 T helper cell, Mast cell, Activated CD8 T cell, CD56bright natural killer cell, Activated B cell, Activated CD4 T cell, Activated dendritic cell, Gamma delta T cell, Immature B cell, Immature dendritic cell, MDSC, Monocyte, Neutrophil, Plasmacytoid dendritic cell, T follicular helper cell, Type 1 T helper cell were decreased between clusterB and clusterA, and Macrophage, Regulatory T cell, CD56dim natural killer cell, Type 17 T helper cell were markedly increased in clusterB compared with clusterA. These results revealed that changes of immune infiltration cells may be involved in the progression of CAD.

### Verification of two molecular subtypes

**Fig. 5:**
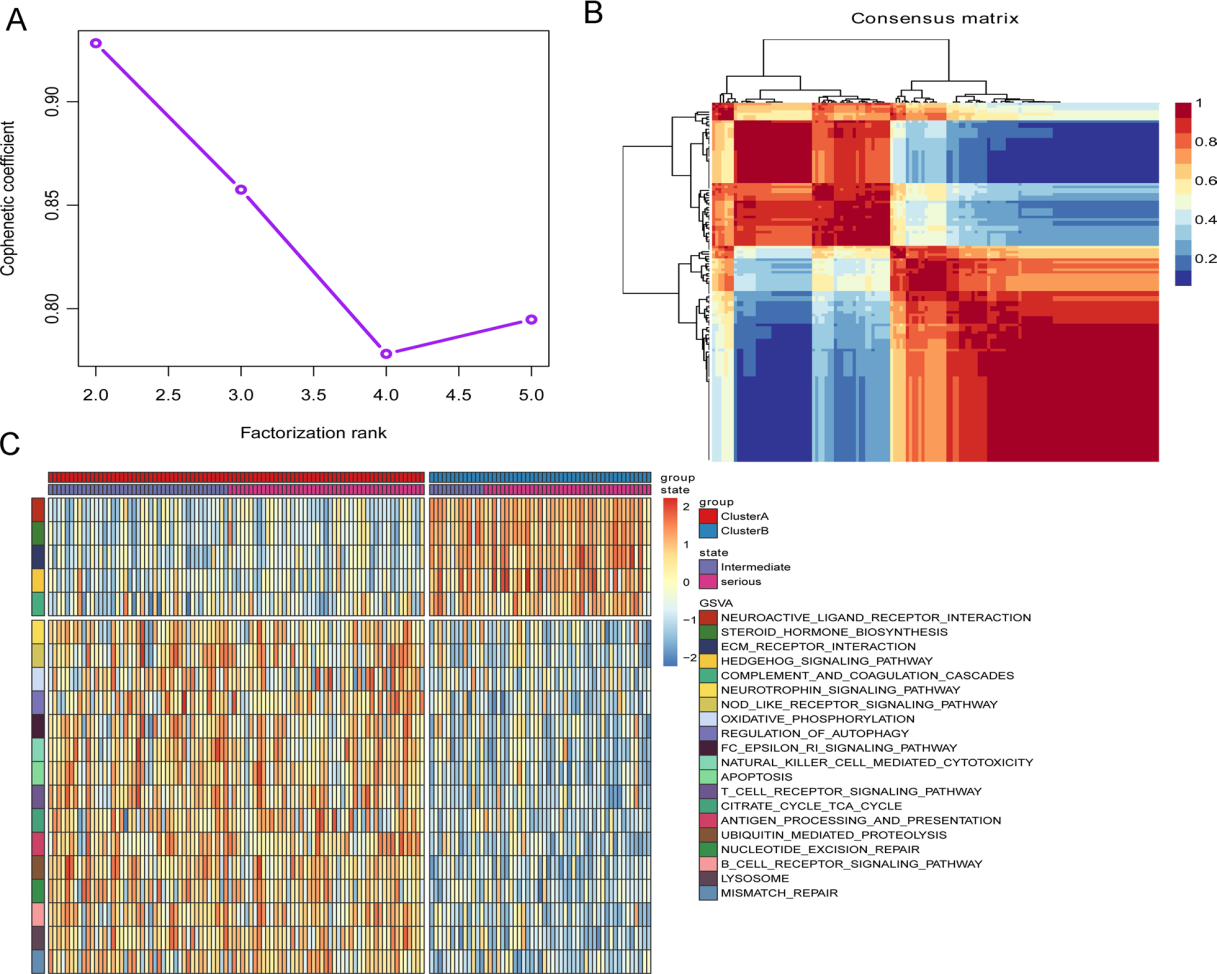
Verification of consensus clusters by ferroptosis-related genes. **A** The relationship between cophenetic and silhouette coefficients with respect to the number of clusters. **B** Consensus matrix heat map for k = 2. **C** GSVA analysis

To further support our findings, we performed NMF clustering on 143 CAD patients in the validation set. When starting from k = 2, comprehensive correlation coefficient started to decrease (Additional file 2: Fig. S2 and Fig. 5A). The heat map showed a clear and sharp boundary, suggesting stable and robust clustering for the samples (Fig. 5B). Clustering results showed that there were also two different subtypes in the validation cohort. Subsequently, Fisher accurate test was used to compare the proportion of Cases (2) patients in different subtypes, and it was found that the percentage of Cases (2) patients in ClusterB was significantly higher than that in ClusterA (Additional file 3: Fig. S3A). Patients with the clusterB subtype had more severe coronary artery stenosis than those with the clusterA subtype. Moreover, CADi was significantly higher in ClusterB than in ClusterA (Fig. 2A). These results suggest that differences in the molecular subtypes of CAD are primarily driven by the severity of CAD. Gene set variation analysis (GSVA) results showed that immune-related pathways (such as the T cell receptor and B cell receptor pathways) were significantly changed in two subtypes of CAD (Fig. 5C). The above results suggest that the occurrence and development of these two subtypes in CAD patients may be related to immune cell infiltration. In addition, to further study the differences in the signaling pathways between control and different subtypes, GSVA heat map of control, clusterA and clusterB was drawn (Additional file 3: Fig. S3B). The result showed that each pathway had noticeable differences among control, clusterA and clusterB, which provide potential research directions for us to further study the pathological mechanism of CAD.

### Development and validation of diagnostic signature model

**Fig. 6:**
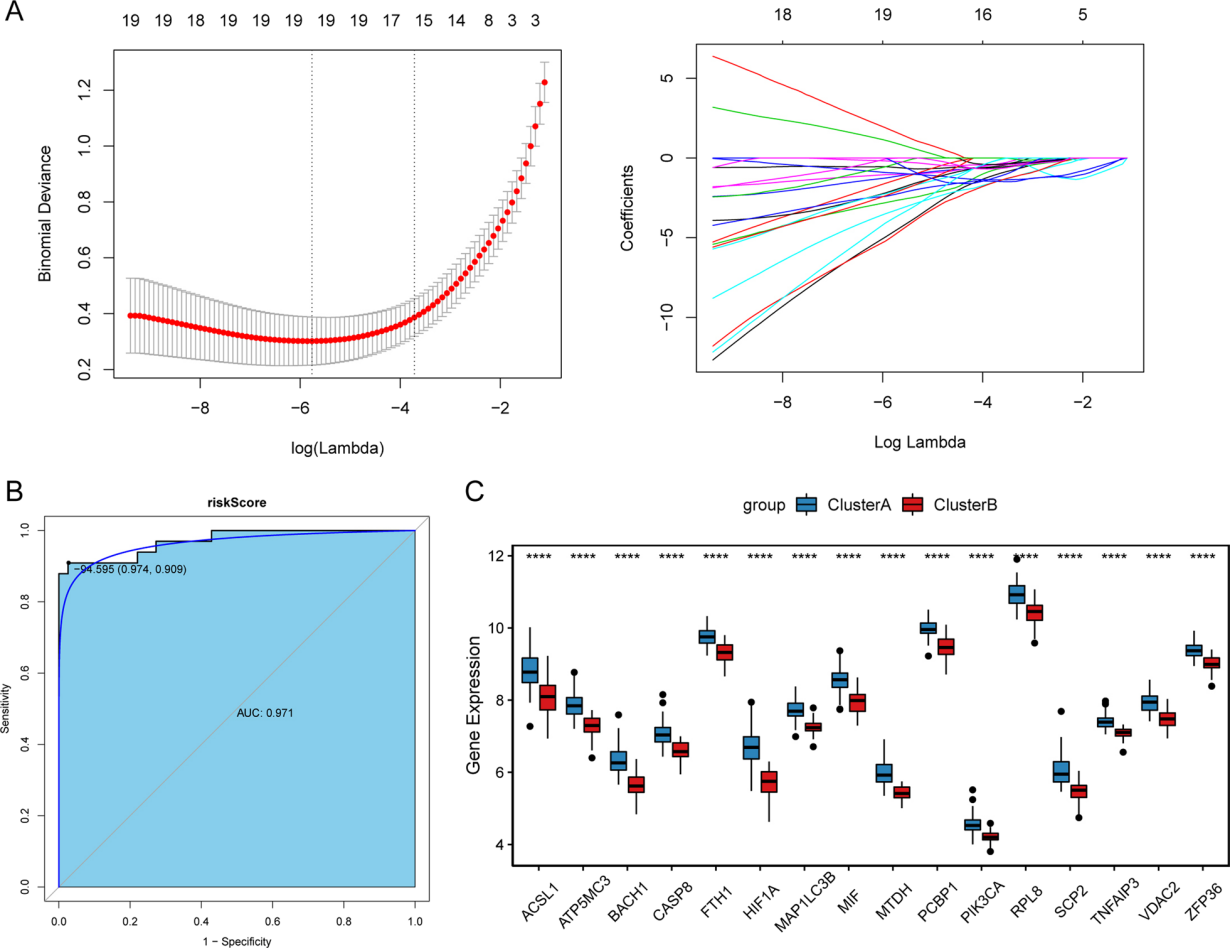
Development of diagnostic signature model. **A** Identification of a 16-gene risk signature by LASSO regression analysis. **B** ROC curves analysis of the training set. **C** The expression levels of these ferroptosis-related genes between two subtypes of CAD

**Fig. 7:**
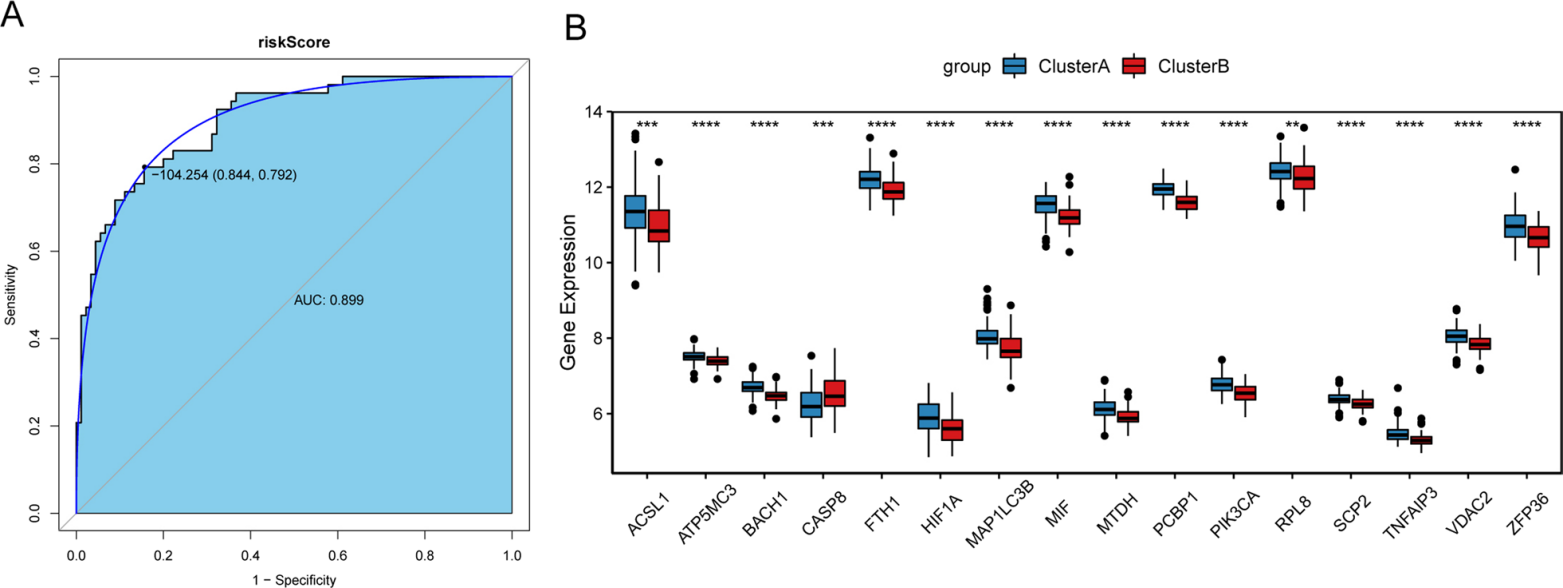
Validation of diagnostic signature model. **A** ROC curves analysis of verification set. **B** The expression levels of ferroptosis-related genes between two subtypes of CAD

**Fig. 8:**
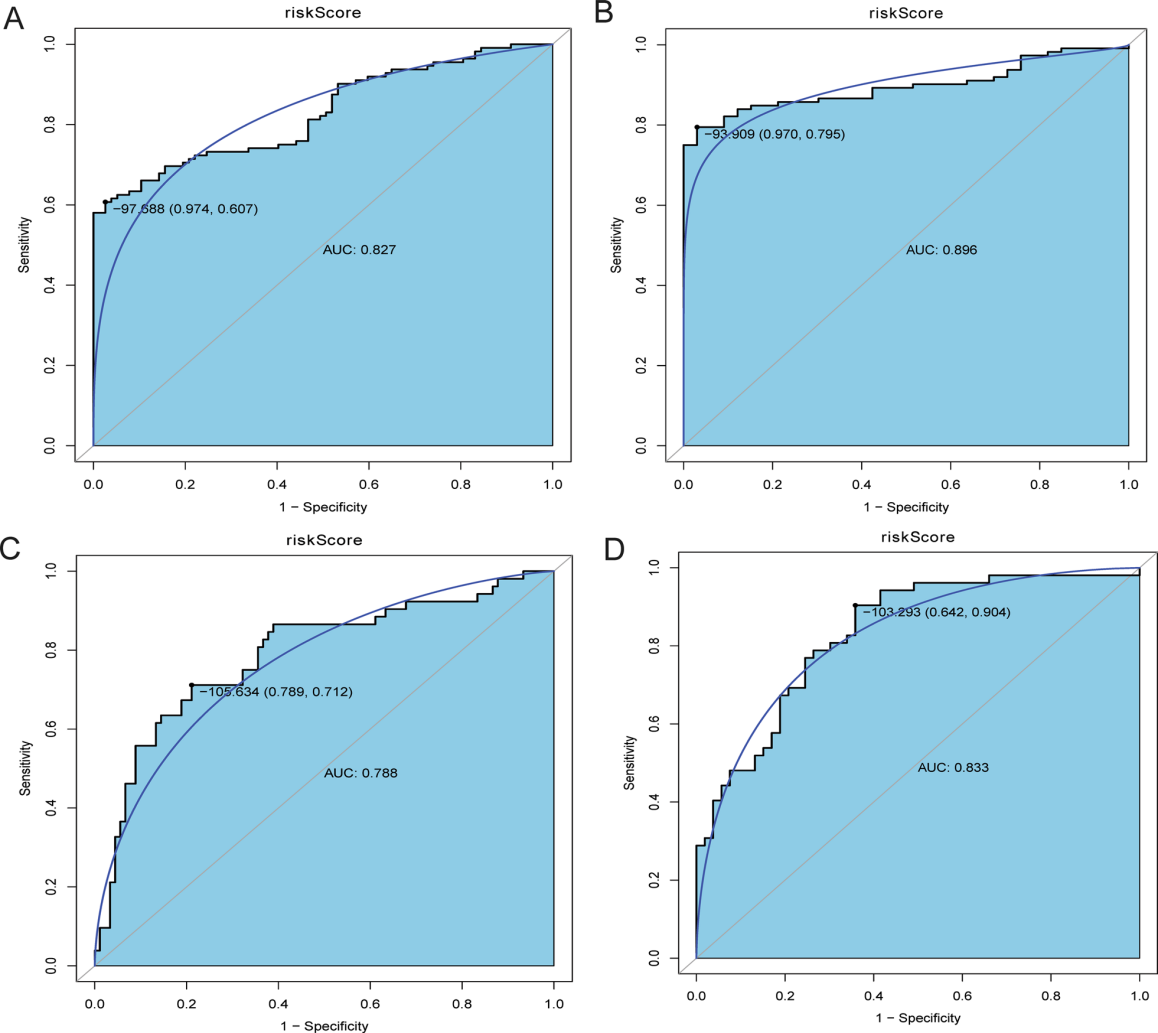
ROC analysis of model between clusterA/clusterB and normal control in GSE12288 and GSE20680 datasets. **A** ROC analysis of model between clusterA and normal control in GSE12288 dataset. **B** ROC analysis of model between clusterB and normal control in GSE12288 dataset. **C** ROC analysis of model between clusterA and normal control in GSE20680 dataset. **D** ROC analysis of model between clusterB and normal control in GSE20680 dataset

CAD were divided into two subtypes based on ferroptosis-related genes. Two subtypes differed in disease progression or severity. Therefore, in order to diagnose different subtypes of CAD, LASSO regression method was used. 16 ferroptosis-related genes (Additional file 9: Table S6) among the 43 differentially expressed ferroptosis-related genes were identified as potential diagnostic markers (Fig. 6A). This diagnostic signature acquired from the training cohort was applied to build the following formula: risk score = (− 1.03*ACSL1) + (− 0.71*ATP5MC3) + (− 0.40*BACH1) + (− 0.39*CASP8) + (− 0.59*FTH1) + (− 1.37*HIF1A) + (− 1.47*MAP1LC3B) + (− 0.52*MIF) + (− 1.47*MTDH) + (− 1.47*PCBP1) + (− 0.15 *PIK3CA) + (− 0.43*RPL8) + (− 0.40*SCP2) + (− 1.20*TNFAIP3) + (− 0.14*VDAC2) + (− 0.67*ZFP36). Next, this model was built in the training cohort to validate its performance. The AUC of this model was 0.971, and the specificity and sensitivity of the model were 97.4% and 90.9%, respectively (Fig. 6B). The expression level of these ferroptosis-related genes between two subtypes of CAD is listed in the Fig. 6C. Results showed that these 16 ferroptosis-related genes were significantly down-regulated in clusterB compared with clusterA. To validate the diagnostic value of the signature in the training cohort, the equation above was used to compute risk score with the gens to gene expression. In the training cohort, the AUC of this model was 0.899, and the specificity and sensitivity of the model were 84.4% and 79.2%, respectively (Fig. 7A). In the training cohort, except for CASP8, the other 15 ferroptosis-related genes were significantly down-regulated in clusterB compared with clusterA (Fig. 7B). These results suggested that these ferroptosis-related genes had high diagnostic value between two subtypes of CAD. In addition, to further analyze the robust of the model in predicting CAD, ROC analyses were performed for different CAD subtypes and normal control (Fig. 8). Results showed that all AUC values were more than 0.7. These results indicate that the diagnostic model can distinguish clusterA and clusterB from the control.

## Discussion

CAD is a heart disease with high morbidity and mortality caused by atherosclerosis [19]. Normally, molecular abnormalities in cardiovascular disease occur before tissue abnormalities [20]. In this study, we obtained 156 ferroptosis-related genes in CAD. Then, CAD samples were clustered into two molecular subtypes based on the expression of 156 ferroptosis-related genes. In addition, DEGs and functional enrichment analysis between two subtypes of CAD were analyzed. Furthermore, except for Natural killer cell and Natural killer T cell, 21 other immune infiltration cells were significantly changed between two subtypes of CAD. Finally, we established a ferroptosis-related diagnostic model based on mRNA expression profiles of CAD patients from GSE12288 dataset and validation it in GSE20680 dataset. On the basis of these analyses, we identified the signature of the 16 ferroptosis-related genes, suggesting that ferroptosis-related genes had high diagnostic value between two subtypes of CAD. To the best of our knowledge, this is the first study to investigate the molecular subtypes of CAD based on the expression of ferroptosis-related genes.

Functional enrichment analysis showed that these DEGs between two subtypes of CAD were mainly enriched in immune-related pathways and processes, such as T cell receptor, mTOR, NOD-like receptor and Toll-like receptor signaling pathways. It has been reported that T cell receptor signaling regulates the differentiation, maintenance and function of T cells, affecting their gene expression, metabolism, cell adhesion and migration [21]. The T cell receptor signaling pathway is significantly up-regulated in CAD patients with heart failure compared with CAD patients without heart failure [22]. It has been reported that mTOR signaling pathway plays an important role in monocyte proinflammatory response in patients with CAD [23]. The Toll-like receptor signaling has been shown to be involved in the pathogenesis of CAD [24, 25]. Recently, Toll-like receptor signaling pathway, and NOD-like receptor signaling pathway associated with inflammation may be involved in regulating the progression of CAD [26]. Meanwhile, ssGSEA results indicated that 21 immune infiltration cells were significantly changed between two subtypes of CAD. Based on the above results, we speculate that changes of immune infiltration cells may be involved in the progression of CAD.

Interestingly, we built diagnostic risk signatures based on 16 ferroptosis-related genes (ACSL1, ATP5MC3, BACH1, CASP8, FTH1, HIF1A, MAP1LC3B, MIF, MTDH, PCBP1, PIK3CA, RPL8, SCP2, TNFAIP3, VDAC2 and ZFP36) through LASSO regression method, which could accurately predict the diagnosis of CAD. Acyl-CoA synthetase long-chain family member 1 (ACSL1) is a member of long-chain acyl-CoA synthetase and plays a key role in the synthesis of triglycerides, phospholipids and cholesterol esters and the oxidation of fatty acids [27]. A study has shown that ACSL1 deficiency helps reduce fatty acid oxidation and increase glucose utilization in the heart [28]. BTB and CNC homology 1 (BACH1) is heme-binding transcription factors that regulate oxidative stress and heme and iron-related metabolic pathways [29]. BACH1 is involved in the aggravation of various oxidative stress-related diseases, including ischemic heart disease [30], and whether it is related to ferroptosis remains unclear. Caspase 8 (CASP8) is key regulator in both extrinsic and intrinsic apoptotic pathways. CASP8 is reported to be decreased in patients with stable angina pectoris compared to controls [31]. It is reported that CASP8 polymorphism variation can be used as a biomarker of CAD susceptibility [32]. Ferritin heavy chain 1 (FTH1), a major intracellular iron storage protein, is a substrate of ferroptosis, which emerges to play vital roles in coronary atherosclerotic heart disease [7]. Li et al. have reported that FTH1 is significantly up-regulated in blood samples of coronary atherosclerotic heart disease patients [33]. HIF1A rs2057482 polymorphism is related to the occurrence of coronary heart disease and some metabolic parameters and cardiovascular risk factors [34]. Macrophage migration inhibitory factor (MIF) is a potent pro-inflammatory cytokine that mediates the inflammatory process in atherosclerosis, and the variation of the MIF gene may be related to the occurrence of CAD [35]. It has been reported that inflammatory TNF α induced protein 3 (TNFAIP3) can be used as a biomarker for the diagnosis of CAD [26]. Although in this study we also found that ACSL1, BACH1, CASP8, FTH1, HIF1A, MIF and TNFAIP3 were significantly changed between two subtypes of CAD, the mechanism of their regulation of CAD progression needs to be further studied.

However, there are some limitations in this study. The function of ferroptosis-related genes in CAD and the pathogenesis of two different molecular subtypes of CAD are still unclear. Therefore, more clinical samples and animal model experiments are needed to verify the results of this study.

## Conclusion

In this study, we divided CAD patients into two different molecular subtypes according to the expression level of ferroptosis-related genes. Then, a total of 1944 DEGs was identified. Among which, 43 DEGs were ferroptosis-related genes. Functional enrichment analysis showed that these DEGs between two subtypes of CAD were mainly enriched in immune-related pathways and processes, such as T cell receptor, mTOR, NOD-like receptor and Toll-like receptor signaling pathways. We also found that 21 immune cells were significantly changed between two subtypes of CAD. The LASSO method was performed to identify and construct a 16 ferroptosis-related genes-based diagnostic signature. Diagnostic efficiency of diagnostic signature measured by AUC in the training set and validation cohort was 0.971 and 0.899, respectively. This study contributes to a more comprehensive understanding of the mechanism of ferroptosis-related genes in CAD, and provides valuable information for further research on the pathogenesis of CAD.

## Supplementary Information

**Additional file 1. Fig. S1.** The relationship between cophenetic, dispersion, evar, residuals, rss and silhouette coefficients with respect to number of clusters in GSE12288 dataset.

**Additional file 2: Fig. S2**. The relationship between cophenetic, dispersion, evar, residuals, rss and silhouette coefficients with respect to number of clusters in GSE20680 dataset.

**Additional file 3: Fig. 3**. Fisher accurately tested the proportion of Cases (2) in different subtypes (A) and GSVA analysis of Control, ClusterA and ClusterB (B).

**Additional file 4: Table S1**. List of 156 ferroptosis-related genes.

**Additional file 5: Table S2**. Differentially expressed ferroptosis-related genes between in two different subtypes of CAD based on "limma" R package

**Additional file 6: Table S3**. All Gene Ontology (GO) terms

**Additional file 7: Table S4.** All signal pathways obtained by gene set enrichment analysis (GSEA).

**Additional file 8: Table S5.** Statistical analysis of immune cell infiltration in different subgroups based on single-sample gene set enrichment analysis (ssGSEA).

**Additional file 9: Table S6.** Ferroptosis-related genes identified by least absolute shrinkage and selection operator (LASSO) regression.

## Data Availability

The datasets used and analyzed during the current study are available from public database Gene Expression Omnibus repository. Accession numbers of the datasets used in the current study are GSE12288 and GSE20680 in Gene Expression Omnibus (https://www.ncbi.nlm.nih.gov/geo).
