# Supplementary figures and images for: Construction and validation of molecular subtypes of coronary artery disease based on ferroptosis-related genes
Source: BMC Cardiovasc Disord. 2022 Jun 22;22:283. doi: 10.1186/s12872-022-02719-1 (PMC9219127; doi:10.1186/s12872-022-02719-1)

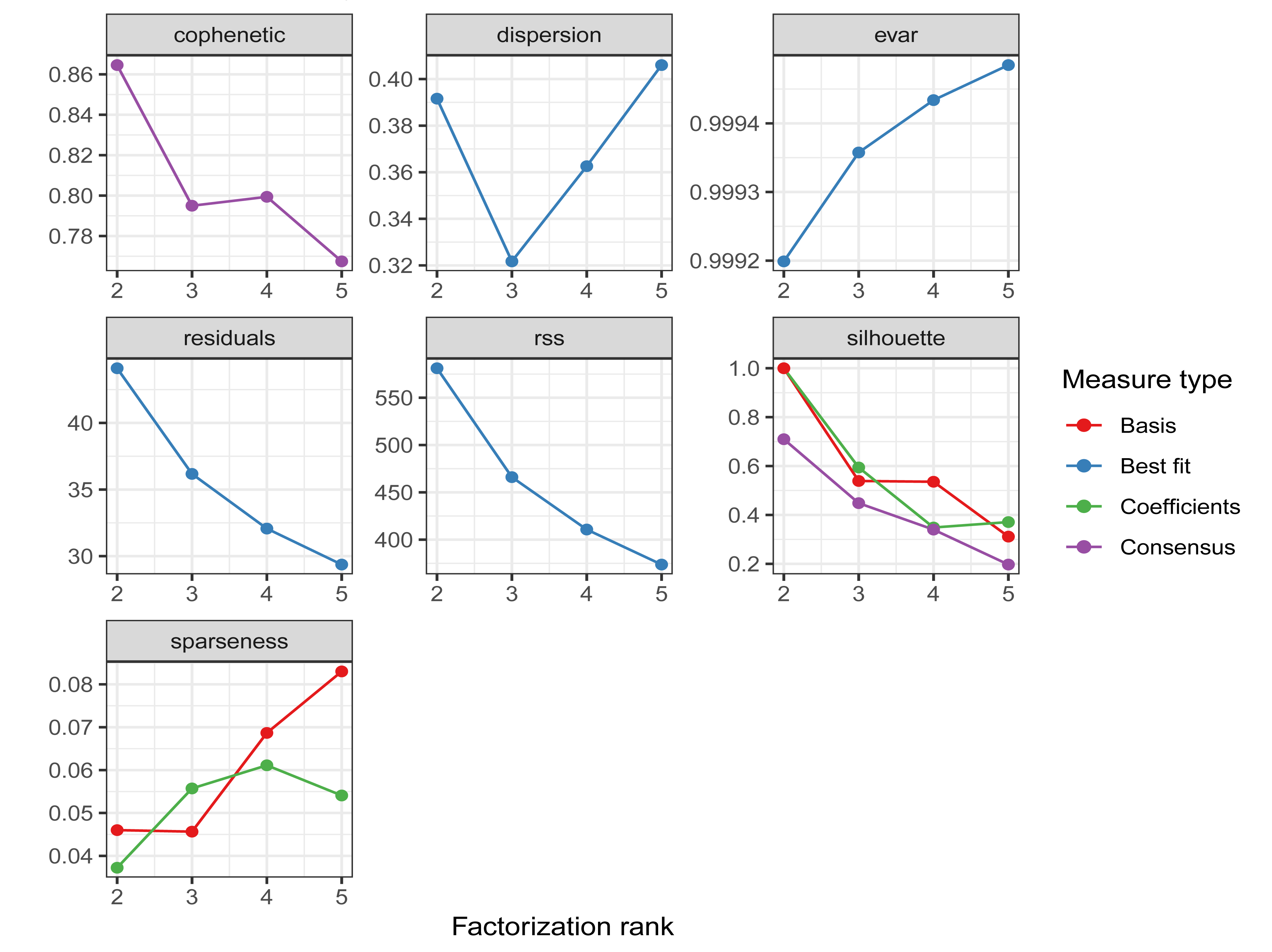

Supplement: Supplementary file 1 — Additional file 1. Fig. S1. The relationship between cophenetic, dispersion, evar, residuals, rss and silhouette coefficients with respect to number of clusters in GSE12288 dataset. [file 12872_2022_2719_MOESM1_ESM.tif]

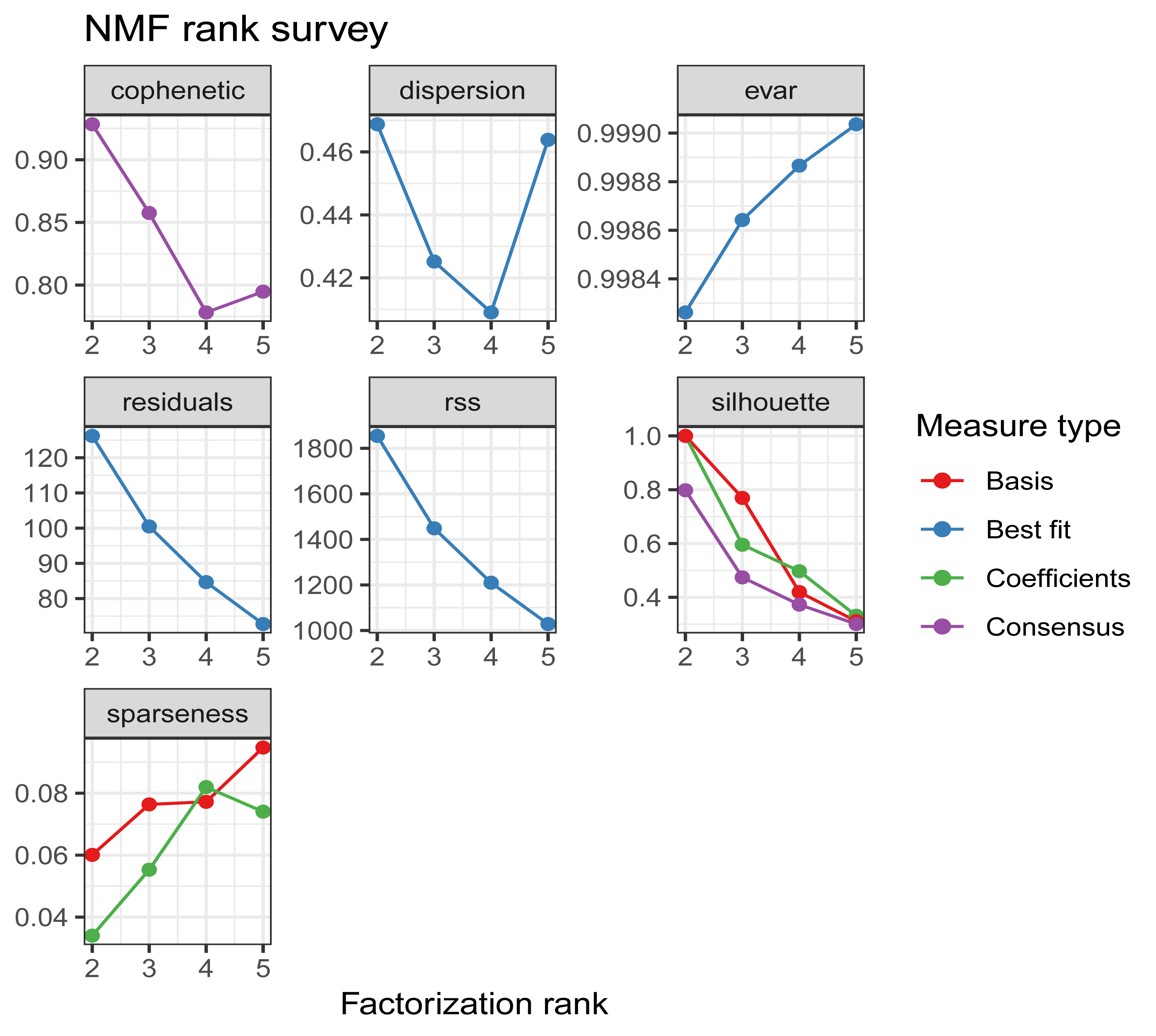

Supplement: Supplementary file 2 — Additional file 2: Fig. S2. The relationship between cophenetic, dispersion, evar, residuals, rss and silhouette coefficients with respect to number of clusters in GSE20680 dataset. [file 12872_2022_2719_MOESM2_ESM.tif]
